# MR-compatible, 3.8 inch dual organic light-emitting diode (OLED) in-bore display for functional MRI

**DOI:** 10.1371/journal.pone.0205325

**Published:** 2018-10-11

**Authors:** YunKyoung Ko, Seong Dae Yun, Suk-Min Hong, Yonghyun Ha, Chang-Hoon Choi, N. Jon Shah, Jörg Felder

**Affiliations:** 1 Institute of Neuroscience and Medicine—4, Forschungszentrum Juelich, Juelich, Germany; 2 Faculty of Medicine, Department of Neurology, RWTH Aachen University, JARA, Aachen, Germany; Universitat Duisburg-Essen, GERMANY

## Abstract

**Purpose:**

Functional MRI (fMRI) is a well-established method used to investigate localised brain activation by virtue of the blood oxygen level dependent (BOLD) effect. It often relies on visual presentations using beam projectors, liquid crystal display (LCD) screens, and goggle systems. In this study, we designed an MR compatible, low-cost display unit based on organic light-emitting diodes (OLED) and demonstrated its performance.

**Methods:**

A 3.8” dual OLED module and an MIPI-to-HDMI converter board were used. The OLED module was enclosed using a shielded box to prevent noise emission from the display module and the potentially destructive absorption of high power RF from the MRI transmit pulses. The front of the OLED module was covered by a conductive, transparent mesh. Power was supplied from a non-magnetic battery. The shielding of the display was evaluated by directly measuring the electromagnetic emission with the aid of a pickup loop and a low noise amplifier, as well as by examining the signal-to-noise ratio (SNR) of phantom MRI data. The visual angle of the display was calculated and compared to standard solutions. As a proof of concept of the OLED display for fMRI, a healthy volunteer was presented with a visual block paradigm.

**Results:**

The OLED unit was successfully installed inside a 3 T MRI scanner bore. Operation of the OLED unit did not degrade the SNR of the phantom images. The fMRI data suggest that visual stimulation can be effectively delivered to subjects with the proposed OLED unit without any significant interference between the MRI acquisitions and the display module itself.

**Discussion:**

We have constructed and evaluated the MR compatible, dual OLED display for fMRI studies. The proposed OLED display provides the benefits of high resolution, wide visual angle, and high contrast video images during fMRI exams.

## Introduction

Functional magnetic resonance imaging (fMRI) has been widely used in brain activation studies. Many fMRI paradigms rely on a visual presentation device either for stimulation of the visual cortex or for presenting elaborate tasks to the subjects, e.g. [1, 2]. In recent decades, there have been significant technical advances in display devices relating to screen size, brightness, and resolution. As a result, their use for supplying high quality stimuli in fMRI has gained increased interest [3].

Conventionally, a shielded LCD television (TV) screen or a beam projector have been used to display paradigms for fMRI studies [4–10]. LCD TVs are advantageous as they are relatively cheap, even for large screen sizes, but have the disadvantage of offering only a limited contrast and a relatively slow response time [4–6]. In addition, due to the effects of aging and changes in ambient temperature, a feedback control is required to maintain constant brightness [4].

As alternatives to TV screens, beam projectors can also be used for the purpose of visual presentations in fMRI. Beam projectors are usually installed outside the MR Faraday shield and commonly use a projection screen inside the magnet bore [7–9]. However, in contrast to LCD based implementations, projectors have a limited brightness, which is often further affected by the poor illumination of the surrounding environment [8–10]. As with the LCD screen, brightness and colour temperature change with the temperature of the projector’s light bulb.

Recently, organic light-emitting diode (OLED) displays have found wide applications due to their superior performance, e.g. in smartphones, large screen TVs, and medical display equipment [11]. The performance advantages of OLED displays include a high contrast ratio, fast response time, true black state, and a slim size [12, 13]. Furthermore, the bendable property of OLEDs [14, 15] potentially allows installation of the display along the bore liner of a scanner. In a combined EEG-fMRI study, OLED displays using a goggle system are expected to provide a stable experimental environment, which was not achieved with a convention stimulus presentation method [16].

In this work, we designed and implemented an MR compatible display using a 3.8” dual OLED. The display was located above a 32-channel receive coil inside the bore of a 3 T clinical MRI scanner. This allowed the screen to be directly viewed by the subject, and was theoretically confirmed by computing the visual angle (the angle a viewed object subtends at the eye) of the display. We investigated its performance and compatibility with MRI under RF irradiation with a body coil. In order to evaluate its impact on the MR receive signal-to- noise ratio (SNR), noise measurements using a spectrum analyser were performed and phantom images were acquired. Finally, an fMRI experiment with a healthy volunteer was carried out, and the feasibility of using the OLED display to present a visual stimulus was proven. An initial report of this work using a different hardware implementation has been presented at the Joint Annual Meeting of the ISMRM-ESMRMB [17].

## Display technology

Visual task presentations require stable display characteristics and optimally a confined environment to exclude sources of data corruption. It has been shown that goggle systems provide more robust stimulation presentation in fMRI compared to conventional screen as they cover a larger visual angle (which increases the number of activated voxels) as well as provide a more direct visual path [7]. However, the technical specifications of the display itself–the most prominent factors being luminance contrast and colour gamut–may also affect the stimulation properties.

It has been shown that changes in luminance modulate visual evoked potentials (VEP) [18] in the primary visual cortex (V1) and increase VEP latency [19]. The changes in blood oxygen level dependent (BOLD) contrast with luminance have been investigated in [20]. Thus luminance levels of visual stimulation devices should be monitored to allow for reproducibility. It must be noted that OLEDs have a lower black luminance compared to LCDs as no backlight is required, thus enabling a higher black-white contrast ratio [21]. Despite several orders of magnitude in contrast differences Stevens’ psychophysical power law suggests only minor differences in perceived contrast between the two types displays [22]. In contrast, a similar level of perceived luminance is encountered at physically different luminance levels for LCDs and OLEDs [21]. Finally, it should be emphasized that luminance depends on the average picture level in OLEDs as power consumption in these devices varies with the number of active diodes and is usually limited by the power supply unit. This is in contrast to LCDs where luminance is governed by the level of backlighting. An extensive treaty on the perception of colour and luminance is given in [23]. This connection is important as OLEDs and LCDs cover different areas of the colour gamut and brightness perception depends on the wavelength of the colour displayed [24]. This aspect of the Helmholtz-Kohlrausch (HK) effect has been demonstrated in fMRI experiments [23] where figures of identical luminance but different colour caused different levels of BOLD activation in V1.

## Methods

### A. General description of the OLED display system

A commercially available 3.8” full high-definition (HD) dual OLED module (TF38101A-V0, TOPFOISON, China) was selected for this study. The dimensions of the OLED module were 67.6 mm × 78.95 mm × 1 mm with a resolution of 1080 x 1200 pixels for a single display and 2160 x 1200 pixels for the dual display, a frame rate of 90 Hz and a brightness of 100 cd/m^2^.

**Fig 1:**
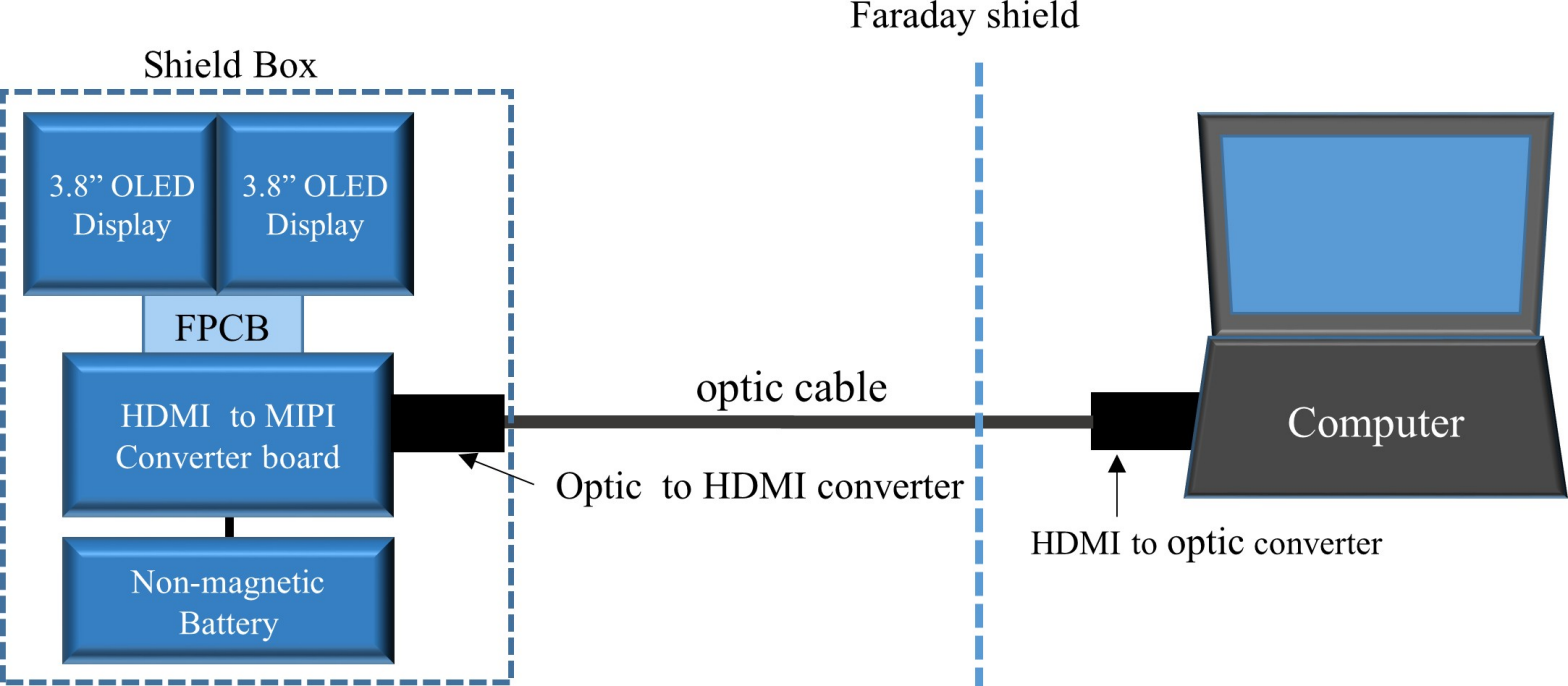
Schematic diagrams describing the connection between OLED display and PC.

The OLED module includes a mobile industry processor interface (MIPI) [25], which is suitable for connecting the dual OLED display to mobile devices, such as smartphones or digital cameras. By default, a personal computer (PC) does not have an MIPI interface but instead uses the high definition multimedia interface HDMI standard. Thus, an MIPI to HDMI converter device (dimension: 12.5 mm × 3.5 mm × 1 mm, TOPFOISON, China) was added to the interface PC and the OLED display. As shown in Fig 1, an HDMI optical transmission system (HD4K-FO-20M-MM, XTENDEX, USA) was used for the connection between the PC and the OLED unit. This minimises potential noise emission from the HDMI connection and has the additional benefit of extending the maximum transmission length. A non-magnetic battery (PGEB-NM5858138-PCB, Powerstream Technology, Utah, USA) was used to power the OLED and converter units. This 6000 mAh battery allows uninterrupted operation of the display for approximately 11 hours. The total cost of the display system components was approximately 650 USD with the OLED itself only accounting for roughly 50 USD. The components associated with the major costs were the HDMI to MIPI converter as well as the optical HDMI transmission system.

### B. MR compatibility issues due to magnetic components

Since all components of the commercially available converter board were not originally manufactured for use inside an MRI scanner, several hardware modifications were required. For example, the switched-mode power supply in the converter board contained ferrite core inductors. The ferrites are saturated in the strong magnetic field of an MRI system and might cause oscillations in the power supply [26]. Furthermore, acquired MR images can be significantly distorted due to the field inhomogeneities caused by the high magnetic susceptibility of ferromagnetic materials [27]. Most importantly, however, using magnetic components is not safe in any MRI environment because of the static force exerted on them. Therefore, the ferrite inductors were replaced with non-magnetic phenolic core inductors (IM-4 series, Vishay Intertechnology, Inc., Pennsylvania, USA).

### C. RF interference and noise issue

**Fig 2:**
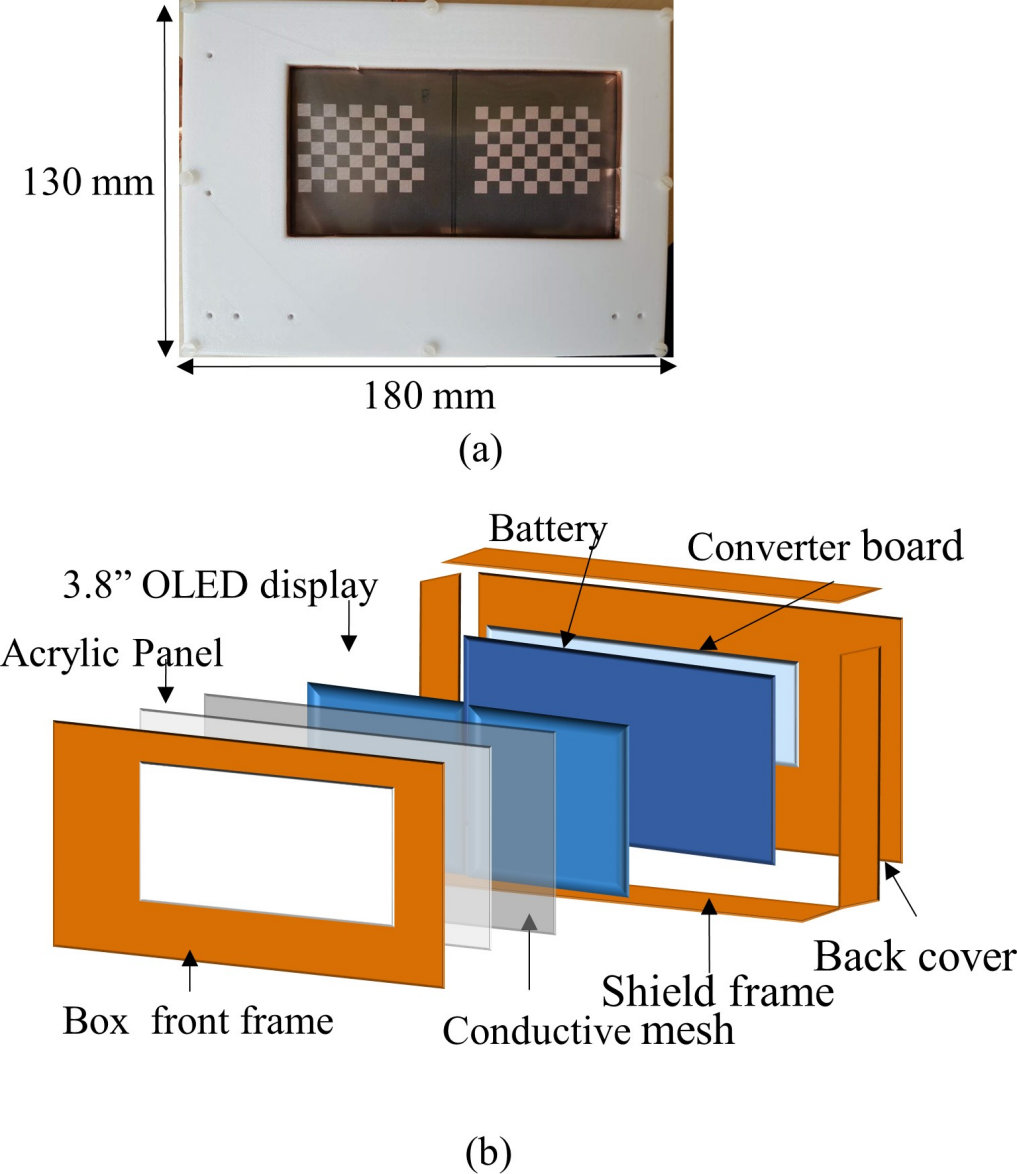
Implemented OLED display. (a) OLED with shield box, and (b) the detailed structures of the shield box. The shield box contains the OLED itself and the video converters without additional shielding between the individual units.

While noise emission from the display module and the converter board may substantially degrade MR image quality, the display is prone to the potentially destructive power from the RF transmit pulses. This can be prevented with the use of a shield box. Fig 2(A) shows the OLED display inside a home-built shield box (dimension: 180 mm × 130 mm × 37 mm). As shown in the explosion plot of Fig 2(B), it includes a transparent acrylic panel in combination with a transparent conductive mesh (8900 Conductive Mesh, Hollandshielding, the Netherlands). The mesh was specified with a broadband shielding effectiveness of 60 dB or better. The plastic housing used to cover the shield box was designed using a commercial CAD tool (Inventor Professional, Autodesk, California, USA) and was built in polycarbonate using a 3D printer (Fortus 400mc, Stratasys, Minnesota, USA). The box was designed to allow for the mechanically stable fixation of all components inside. For shielding, copper tapes (3M, Minnesota, USA) were attached to the inside of the box with a small overlap between neighbouring tape sections.

### D. Noise measurement of the OLED and analysis

**Fig 3:**
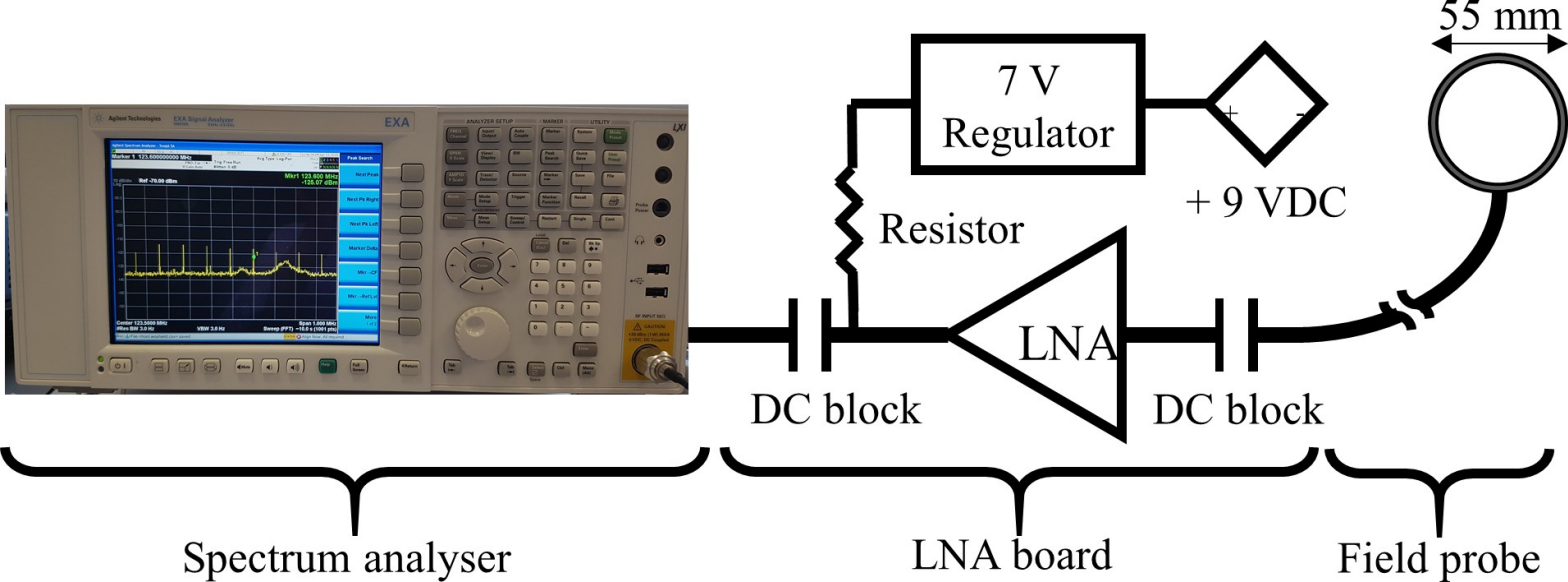
System set-up to measure noise with LNA board and field probe.

The OLED display and the converter board were installed and operated inside the shield box and its shielding effectiveness was evaluated on the bench. For this purpose, a field probe (55 mm diameter) and a low-noise amplifier (LNA) board, shown in Fig 3, were designed and implemented. The amplifier used was a commercial LNA (ERA-4SM+, Mini-Circuits, NY, USA) with a gain of approximately 14 dB at 123.25 MHz. The LNA was supplied via an on-board voltage regulator (Fig 3) in order to reduce any influences from potential noise pickup by the supply lines. Fig 3 shows the system used for noise emission measurements, including the spectrum analyser (N9010A, Keysight Technologies, Santa Rosa, USA). Peak detection was used to accurately measure the noise, and the measurement parameters of the spectrum analyser were as follows: start frequency = 123 MHz, stop frequency = 124 MHz, resolution bandwidth = 3 Hz, visual bandwidth = 3 Hz, number of points = 1001, sweep time = 10.012 seconds. The noise emission of the OLED display was measured with/without the shield cover, while the pickup loop was kept at a fixed distance (3 cm) to the display.

### E. Display properties

Optical measurements of the shielded display were carried out and compared to those obtained from an LC display system which is used routinely for fMRI experiments, e.g. [28–32]. The LCD system consists of a 30” Apple Cinema HD Display (Apple Inc., Cupertino, USA) shielded by a metallic case and a glass panel including a copper mesh (Infratro GmbH, Munich, Germany).

Luminance values were measured for a full white and a full black display using the opticalCAL (Cambridge Research Systems, Rochester, UK) with the displays mounted inside the RF shielded box. Illumination level was measured at a distance of 20 cm for the OLED setup and a distance of 3 m for the LCD screen, which is approximately the distance of the LCD towards the magnet iso-centre in our 3 T MRI suite. Measurements were acquired with a Mavolux 5032b (GOSSEN Foto- und Lichtmesstechnik GmbH, Nürnberg, Germany) and were carried out when displaying a full white screen and in a dark room with no other source of illumination.

### F. Visual angle of the display

**Fig 4:**
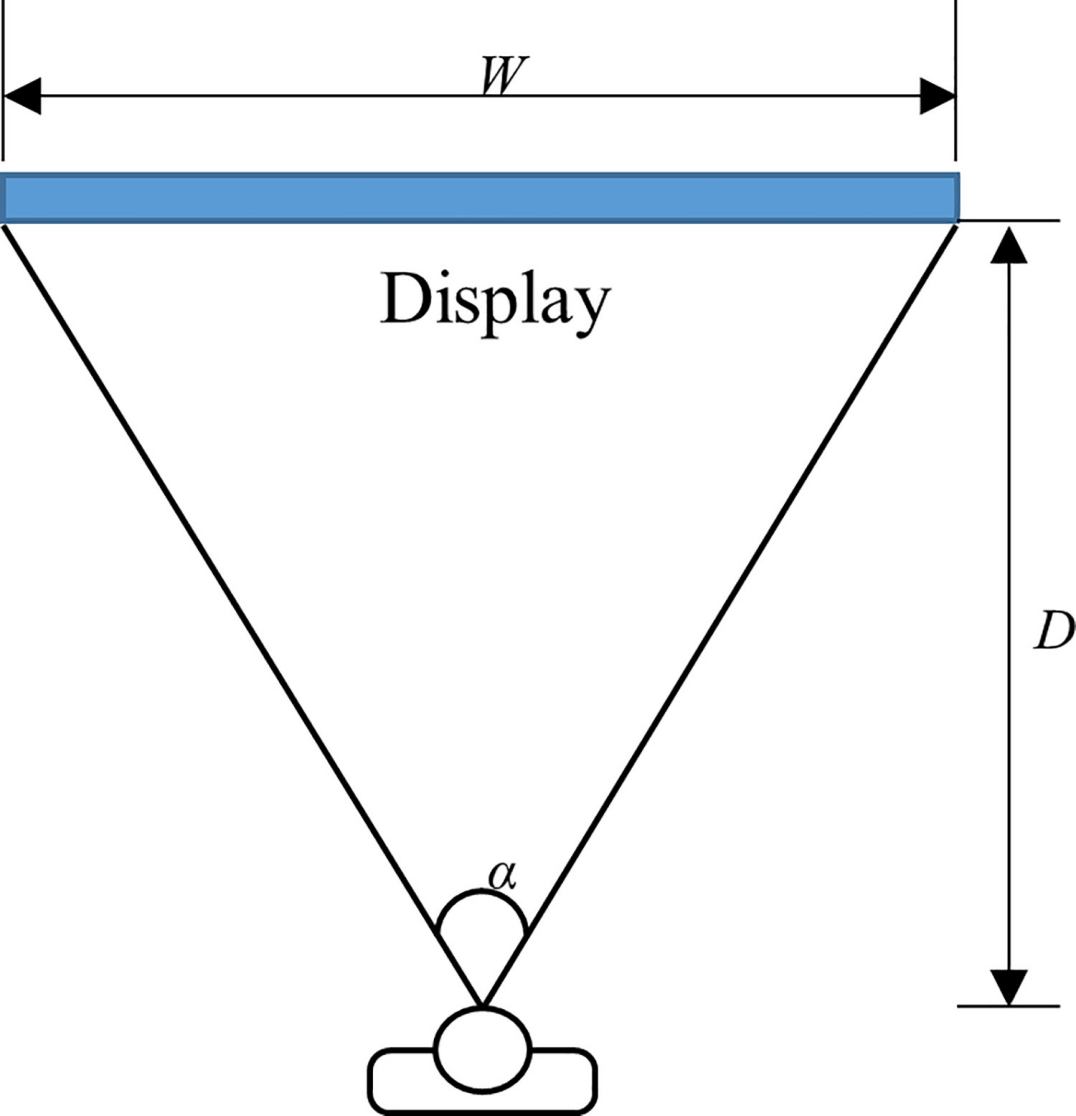
The schematic of visual angle, display width and distance.

Fig 4 shows the relationship between the horizontal visual angle of the display, where W is the width of the display and D is the distance from the subject to the display. This relationship can be derived based on the trigonometric function as below [33]:

$$\alpha =2*\tan^{-1}\left(\frac{W}{2D}\right)$$ (1)

where α denotes the display’s angle of view. Using Eq (1), the visual angle of the OLED display was calculated and compared to those of a display located outside the bore in order to evaluate potential image display degradation at the periphery as well as confinement of the fMRI experimental environment [16]. For comparison, two currently commercially available screen sizes were considered: a 32-inch LCD display–the largest display size of the BOLDscreen (Cambridge Research Systems Ltd., UK)–and a 40-inch LCD display (UN40MU6300, Samsung, Suwon, Korea).

### G. System connection and setup in MRI system

**Fig 5:**
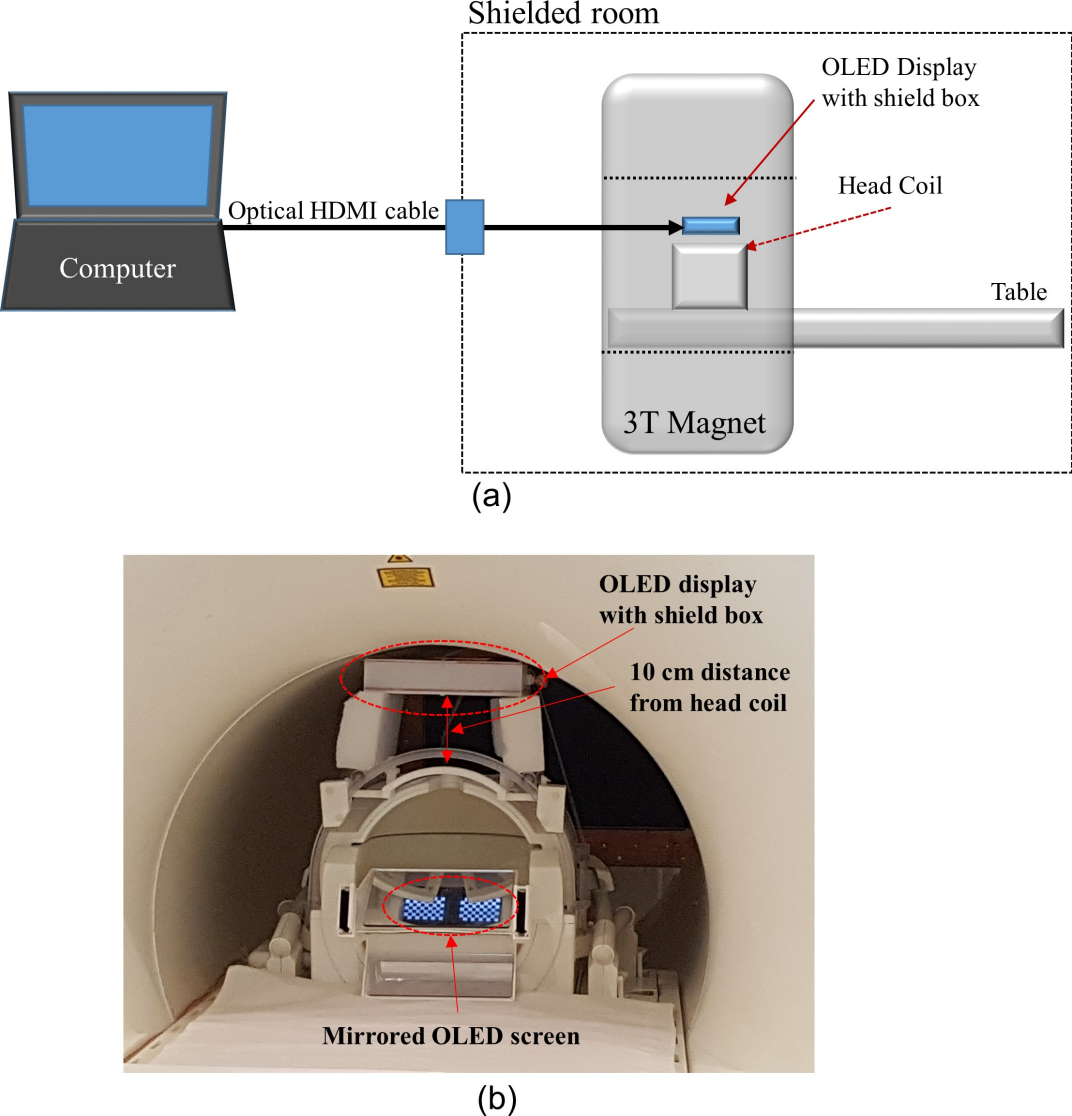
The installation of the OLED display inside bore. (a) A schematic diagram and (b) a photo of the OLED display installed on the head coil.

The general setup of the OLED display unit and its connection to the PC is shown in Fig 5(A) and its mounting inside the bore is depicted in Fig 5(B). The OLED display was fixed to a commercial 32-channel head receive array coil (Siemens Healthineers, Erlangen, Germany), located inside of the magnet bore on the patient table. The minimum distance from the head coil to the display screen was kept at 10 cm to ensure that the subject could comfortably look at the screen. If the distance becomes shorter, the subject’s field-of-view (FOV) covers only a part of the display area, due to the wide visual angle. A larger distance, such as the 30 cm described in [21], is feasible and facilitates display fixation but requires a more sophisticated experimental setup for sturdiness. With the chosen setup, the display can be observed directly without requiring a mirror or optical lenses.

### H. MR based validation

To inspect the impact on receive SNR, MR images were acquired with the OLED displaying a full HD video and with the unit powered off. All scans were performed on a MAGNETOM Tim Trio 3 T MRI scanner (Siemens Healthineers, Erlangen, Germany) using a 32-channel head receive array coil. The phantom employed was a 170 mm spherical water phantom doped with NiSO_4_ x 6H_2_O (Siemens Healthineers, Erlangen, Germany). Data were acquired using a gradient echo (GRE) sequence and the imaging parameters of the pulse sequence were as follows: repetition time (TR) = 40 ms, echo time (TE) = 3.84 ms, number of slices = 1, slice thickness = 5 mm, matrix size = 128 × 128, bandwidth (BW) = 260 Hz/Px, and flip angle (FA) = 25°. Noise measurements for the SNR calculation used the same sequence and parameter, with the transmit power set to zero. Both phantom and noise images were reconstructed using the sum of squares and the SNRs were then calculated according to the NEMA2 method [34]. For the calculation of SNRs, noise standard deviation values were corrected for the non-Gaussian distribution of magnitude data according to [35] (the standard deviation was divided by a correction factor of 0.71).

To verify the applicability of the OLED display for fMRI, a visual functional study was performed. It used a visual checkerboard paradigm to activate the visual cortex. A simple block paradigm was designed where a black and white checkerboard, alternating at a frequency of 8 Hz, was interleaved with low-level baseline phases. Three dummy scans were used to reach a steady state, and then 72 scans, comprising six cycles of baseline-activation, were applied to acquire fMRI data, where each state (i.e. baseline or activation) consisted of 6 scans. A healthy male volunteer (age: 41 years) participated in the study. Before scanning, written informed consent was obtained from the subject. The local ethics committee (RWTH Aachen University, Germany) approved the study protocol, screening questionnaires, and consent forms.

fMRI data were acquired with the following imaging parameters: FOV = 200 × 200 mm^2^, TR/TE = 2000/30 ms, number of slices = 33 with 3 mm thickness, matrix size = 64 × 64, BW = 2232 Hz/Px and FA = 90°. Additionally, to normalise individual data to a standard space, a 3D, high-resolution image was collected with a T_1_-weighted, magnetisation-prepared, rapid acquisition gradient echo (MPRAGE) pulse sequence using the following parameters: FOV = 256 × 256 × 176 mm^3^, TR/TE = 2250/3.03 ms, matrix size = 256 × 256, flip angle = 9°, GRAPPA factor of 2 with 64 auto calibration signal lines and 176 sagittal slices with 1 mm slice thickness.

The acquired fMRI data were analysed using SPM12 (Wellcome Department of Imaging Neuroscience, UCL, London, UK). Following the pre-processing of the fMRI data (realignment, co-registration with MPRAGE, normalisation to the MNI space and spatial smoothing with a Gaussian kernel), the first level analysis was applied with a GLM model. After estimating the GLM parameters with the ReML method, a contrast image was collected with a contrast vector of [-1 (baseline); 1 (activation)] [31].

## Results

The MR-compatible OLED display designed and presented here operated well in the MR environment without any visible image distortions or interruption of the full HD video transmission, as observed while running the GRE and echo-planar imaging sequences described above. Neither gradient switching nor RF emission from the body coil caused visible effects on the video display.

**Fig 6:**
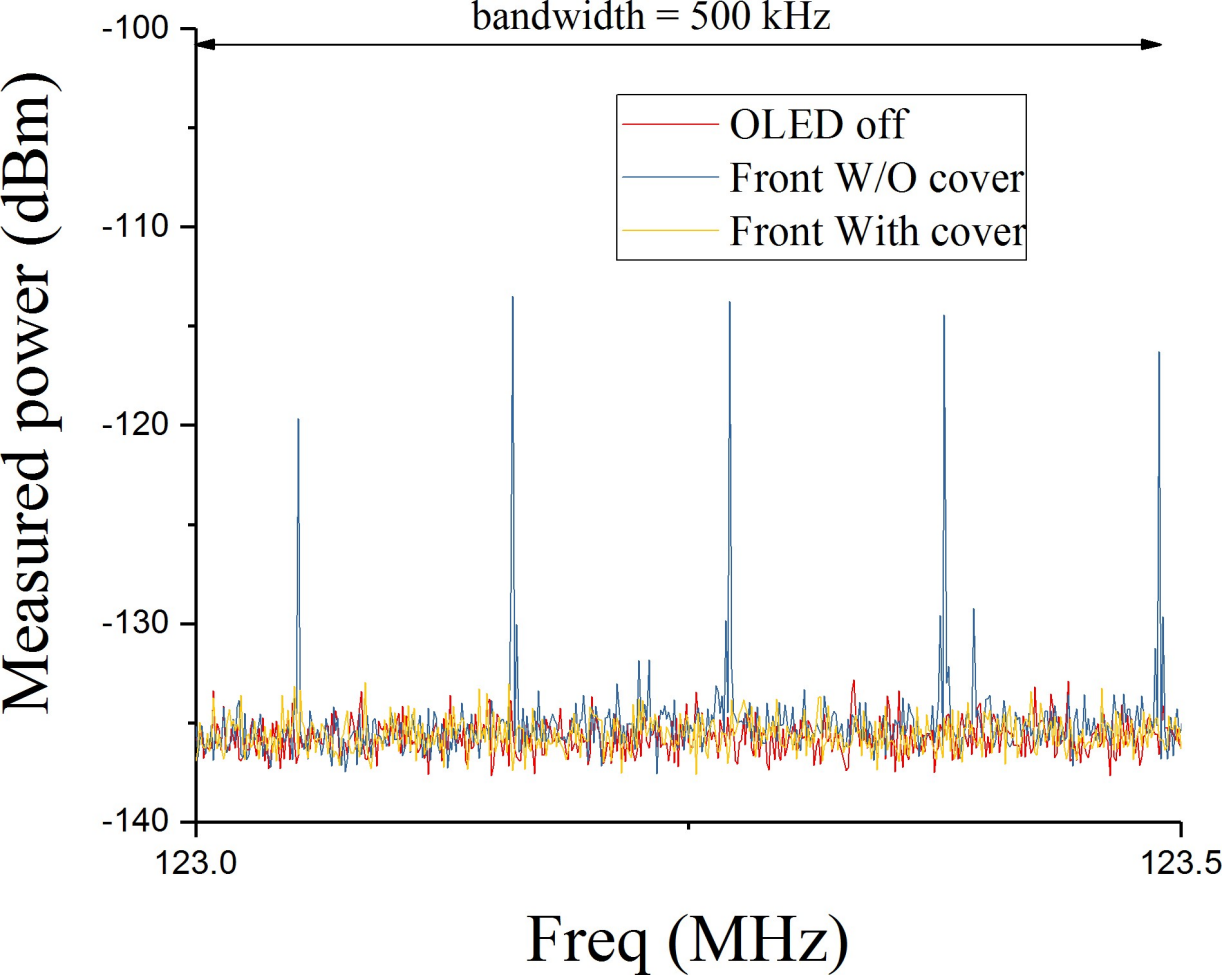
Measured noise emission of the OLED display. The display is cantered on the Larmor frequency of the MRI system (123.25 MHz) and covers a bandwidth greater than the bandwidth used in the fMRI acquisitions.

Fig 6 shows the front-side noise emission of the OLED display with and without the shield box. The noise spectrum shows a periodic EM emission (blue line) that was suppressed to within the noise floor of the spectrum analyser when shielding was applied (yellow line).

The measured white luminance for a full white screen was 43.6 cd/m^2^ for the OLED. The black luminance was below the measurement system threshold. The LCD achieved a white and black luminance of 105.4 cd/m^2^ and 0.346 cd/m^2^, respectively. This results in a Michelson contrast [36] of 1 for the OLED and of 0.99 for the LCD. Illumination levels were 14.8 lux and 5.2 lux for the OLED and the LCD, respectively.

Because the OLED display is located only a few centimetres away from the eyes of the patient, the visual angle is larger than that of a screen placed behind the scanner bore, as can be seen from the values calculated in Table 1. The visual angle of dual OLED was 34.7°. The visual angles of the 32-inch LCD display were 15.1 and 11.4 degrees, according to the distances 300 cm and 400 cm, respectively. For the 40-inch LCD display, the angles were 17° and 12.8°, according to the distances of 300 cm and 400 cm, respectively. As described in [16] the wider viewing angle helps to maintain a controlled experimental environment in fMRI examinations.

**Table 1:** Calculated visual angle of dual OLED compared to that of commercial screens mounted outside the scanner bore.

	3.8 inch dual OLED	32 inch LCD TV		40 inch LCD TV	
W *[cm]*	12.5	80		90	
*D [cm]*	20	300	400	300	400
*α [degree]*	34.7	15.1	11.4	17	12.8

The single images show the phantom acquisition using the GRE sequence and the corresponding noise scan with transmit power set to zero. The red dashed circle shows the ROI used for SNR computation. Images were reconstructed using sum-of-squares and SNR values were corrected for the non-Gaussian distribution of magnitude data according to [34, 35]–the standard deviation was divided by 0.71.

**Fig 7:**
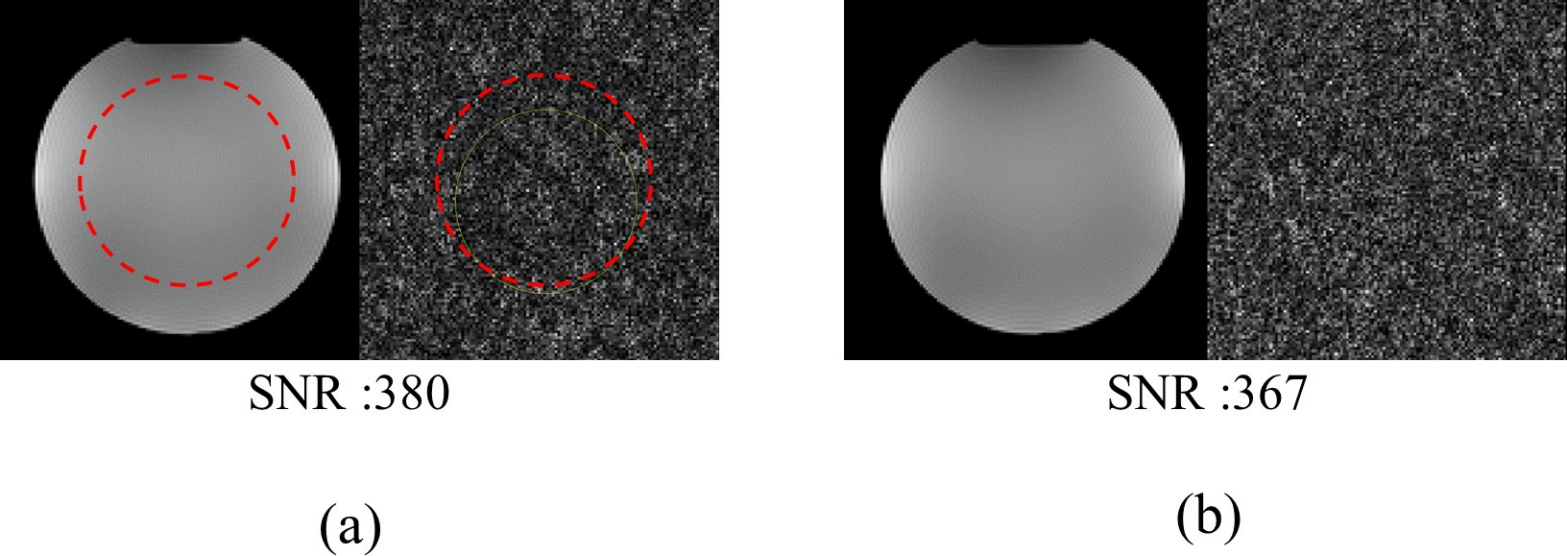
SNR evaluation with the display turned off (a) and the display in operation (b).

Fig 7 shows GRE images obtained with and without operating the OLED display. Compared to the reference case, the SNR reduction caused by powering the OLED display on was only 3% (Fig 7(A)). Visual inspection of the noise image suggests that data were acquired without any noticeable artefacts.

**Fig 8:**
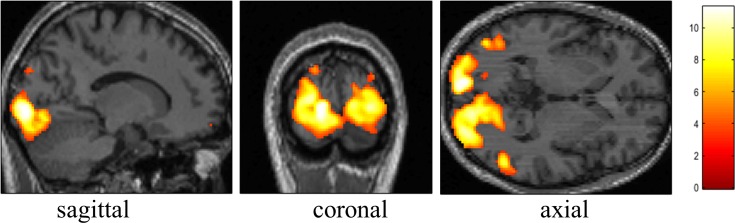
BOLD activation when stimulating the visual cortex using the display presented here and a checker-board block paradigm. Hemispheric asymmetries as observed for LCD activation in volunteers with a dominant eye in [7] are not visible in the activation pattern acquired here.

Fig 8 shows fMRI results for the first level analysis. The activated voxels were obtained with an uncorrected p-value < 0.001 (t-value > 3.73), and are overlaid on the MPRAGE scan. The activation regions for the whole slices are presented in sagittal, coronal and axial views. The sectional views were probed at an MNI coordinate of (x, y, z = -14, -92, 0), to exhibit the maximum t-value. As shown in the Fig 8, visually-induced brain activations were successfully detected around the visual cortex. The results obtained here are in agreement with those in [31] and indicate that the visual stimuli were delivered to the subjects effectively.

## Discussion

We have successfully implemented an MR compatible, 3.8” dual OLED display for fMRI using a commercially available OLED connected via an HDMI-to-MIPI converter to a standard PC. A partially transparent shield box was designed and implemented to reduce noise emission from the display and to prevent potential damage from RF transmit pulses. The system uses an optical transmission system for the HDMI signal that has two advantages over the electrical transmission. First it avoids broadband noise emission, known to arise from skew on differential lines [37], and second, it reduces the risk of heating and the potential for local burns arising from unbalanced currents on the cable shields [38, 39]. A non-magnetic battery was adapted to supply power for the OLED display, making it possible to remove the power cable from the display. This had the benefit of avoiding possible shield currents on the power supply cable as well as mitigating potential noise interference from fluctuation on the power supply. In fact, coupling between RF coil and cable sheets was the main reason why the solution described in [17] was abandoned. Overall, it was demonstrated that the operation of the display system, while transmitting with the body coil and receiving with a highly sensitive multi-channel receive array, does not interfere with MR imaging performance or with video display quality.

The selected OLED is capable of supplying a full HD display resolution with high contrast. The use of the OLED display for fMRI was successfully demonstrated with a visual functional study. The display operates as a second screen for standard PCs and can thus be easily integrated for the presentation of various tasks. During the fMRI test, the subject was able to see the full screen directly, with the added benefit of a wider visual angle and consequently less distraction from the surroundings. This was achieved by mounting the OLED in the subject's direct line of sight with a suitable distance from the head coil. It should be noted that an OLED based goggle system is commercially available but the purpose of this investigation has been to provide a low-cost alternative that can be modified in house for operation in our high field 7 T and 9.4 T scanners.

In the future, we intend to evaluate the performance of this MR-compatible OLED display further by direct comparison with conventional LCD display, and to carry out fMRI experiments with a larger number of subjects to determine effects of stimulus presentation on BOLD contrast as well as long-term stability of the displays when operated in the MR environment. Given the relative lack of publications that couple display properties with variations in fMRI data, this is a highly interesting project. It can also take into account that the blue diode in OLED displays is known to deteriorate faster than the red and green ones [40], thus creating deviations of colour-balance in long-term usage.

With recent reductions in the cost of OLED displays, the system presents an easy implementation of a high-quality display for fMRI experiments. Furthermore, the system presented here–if integrated into a goggle system–can supply virtual reality (VR) and 3D images to subjects [41] by virtue of two independent OLED panels. In contrast to commercial goggle systems, which are costly even at lower resolutions, the presented system shows the potential for implementing a low-cost, advanced, in-bore display unit. Another possible benefit of OLED displays is that they can be curved; allowing the display to be mounted directly onto the magnet bore liner. Recently, a transparent OLED technology [42], and a quantum dot light-emitting diode [43] have been developed and it is possible that this technique could also be applied to MRI in the future.

## Supporting information

S1 File CAD Files. This file contains the CAD files for the shield box.
(ZIP)

S2 File Manufacturing Instructions. Several slides describing the manufacturing of the shielded display.
(PPTX)
