# Supplementary material for: MR-compatible, 3.8 inch dual organic light-emitting diode (OLED) in-bore display for functional MRI
Source: PLoS One. 2018 Oct 11;13(10):e0205325. doi: 10.1371/journal.pone.0205325 (PMC6181352; doi:10.1371/journal.pone.0205325)
Supplement: S2 File — Several slides describing the manufacturing of the shielded display. (PPTX) [file pone.0205325.s002.pptx]

## Slide 1
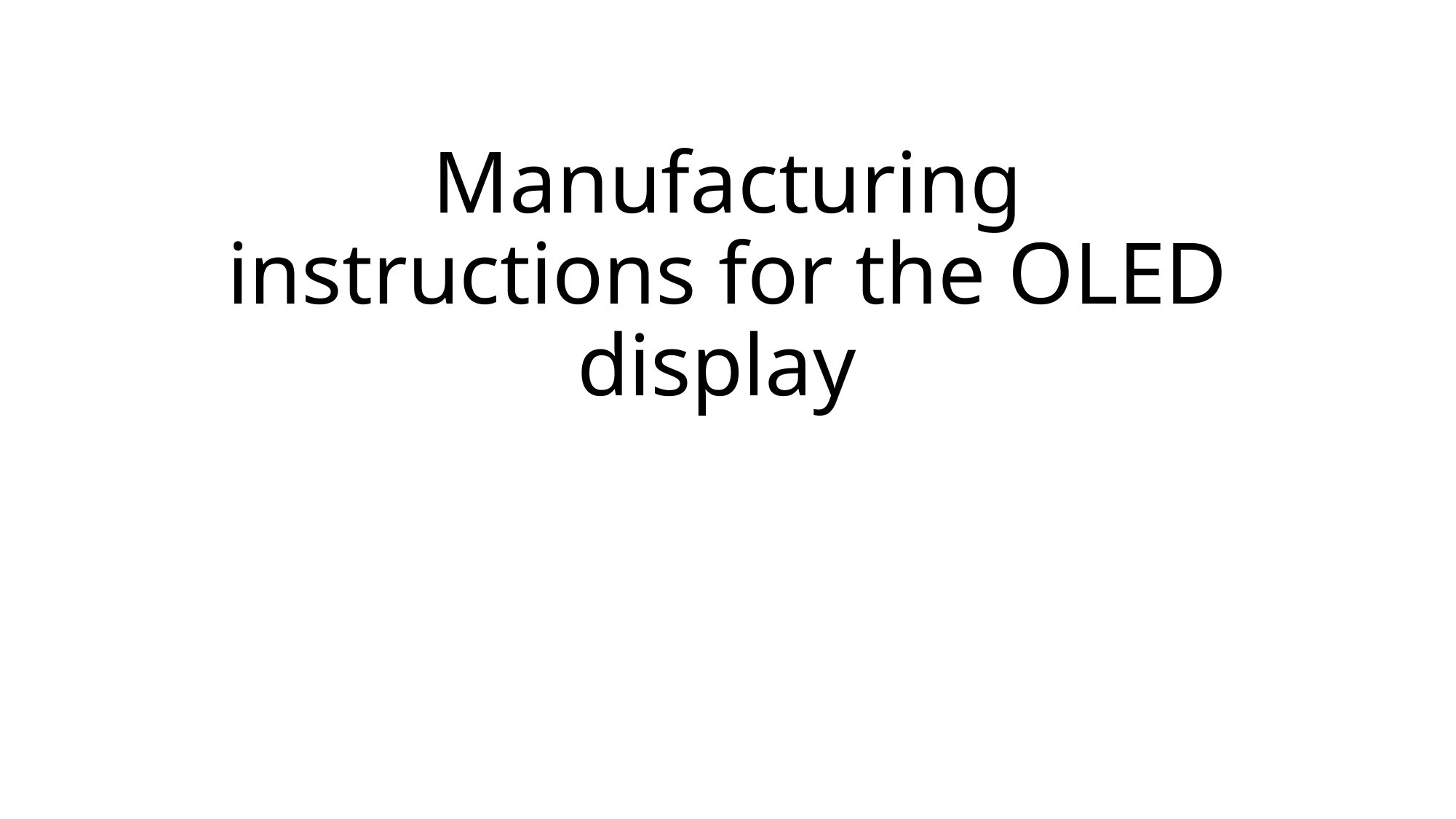

# Manufacturing instructions for the OLED display

## Slide 2
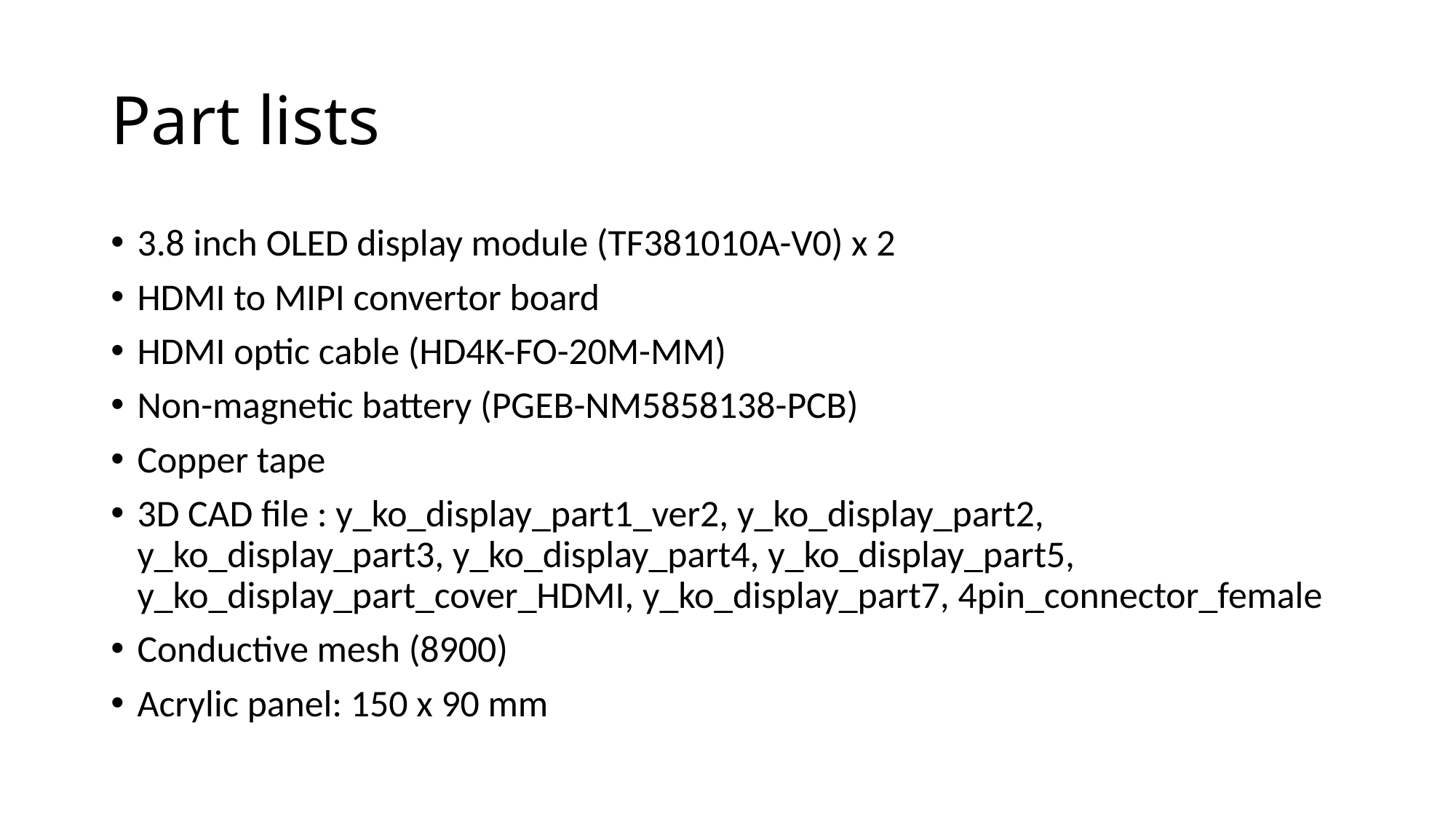

# Part lists
3.8 inch OLED display module (TF381010A-V0) x 2
HDMI to MIPI convertor board
HDMI optic cable (HD4K-FO-20M-MM)
Non-magnetic battery (PGEB-NM5858138-PCB)
Copper tape
3D CAD file : y_ko_display_part1_ver2, y_ko_display_part2, y_ko_display_part3, y_ko_display_part4, y_ko_display_part5, y_ko_display_part_cover_HDMI, y_ko_display_part7, 4pin_connector_female
Conductive mesh (8900)
Acrylic panel: 150 x 90 mm

## Slide 3
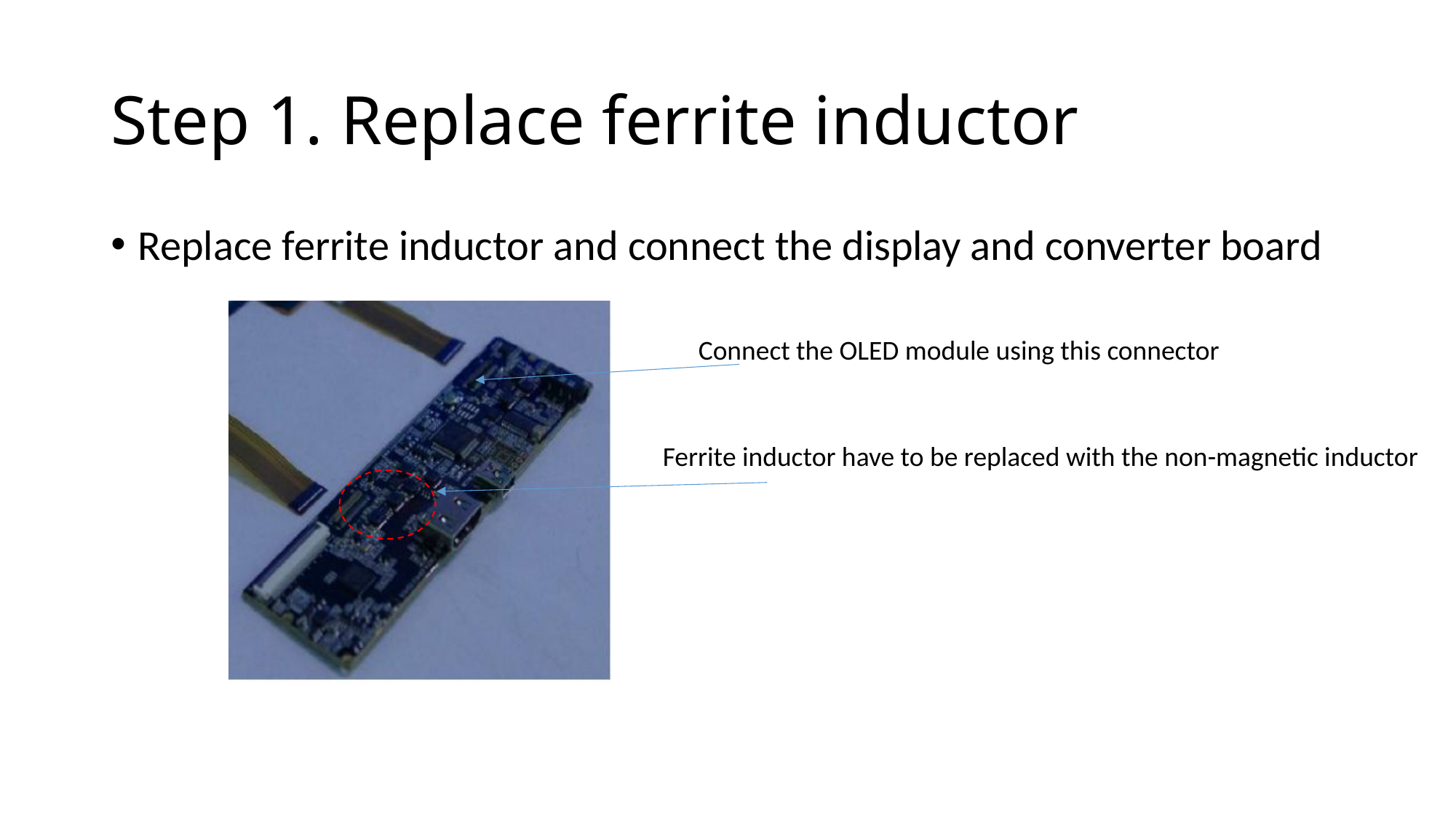

# Step 1. Replace ferrite inductor
Replace ferrite inductor and connect the display and converter board
Connect the OLED module using this connector
Ferrite inductor have to be replaced with the non-magnetic inductor

## Slide 4
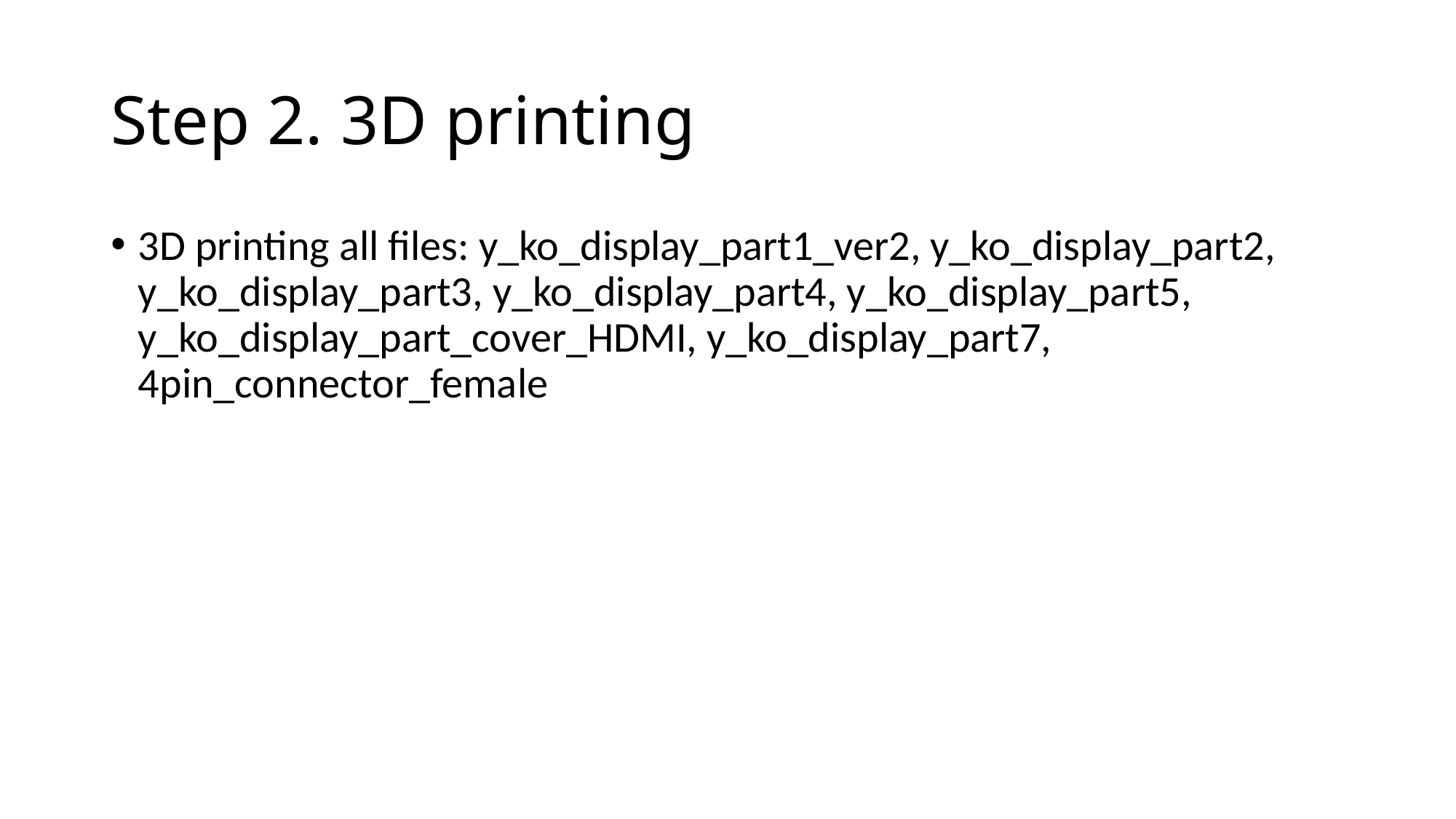

# Step 2. 3D printing
3D printing all files: y_ko_display_part1_ver2, y_ko_display_part2, y_ko_display_part3, y_ko_display_part4, y_ko_display_part5, y_ko_display_part_cover_HDMI, y_ko_display_part7, 4pin_connector_female

## Slide 5
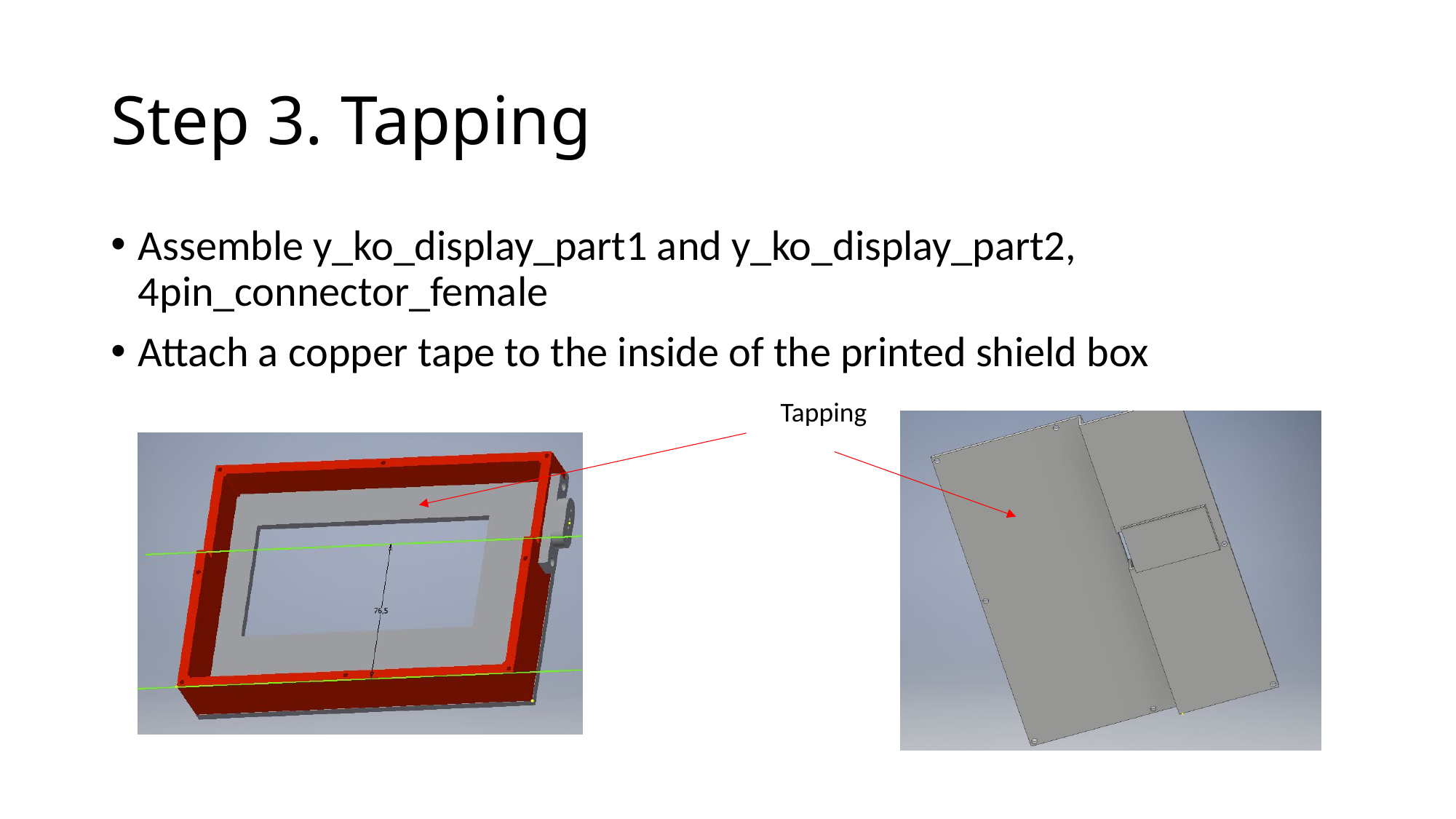

# Step 3. Tapping
Assemble y_ko_display_part1 and y_ko_display_part2, 4pin_connector_female
Attach a copper tape to the inside of the printed shield box
Tapping

## Slide 6
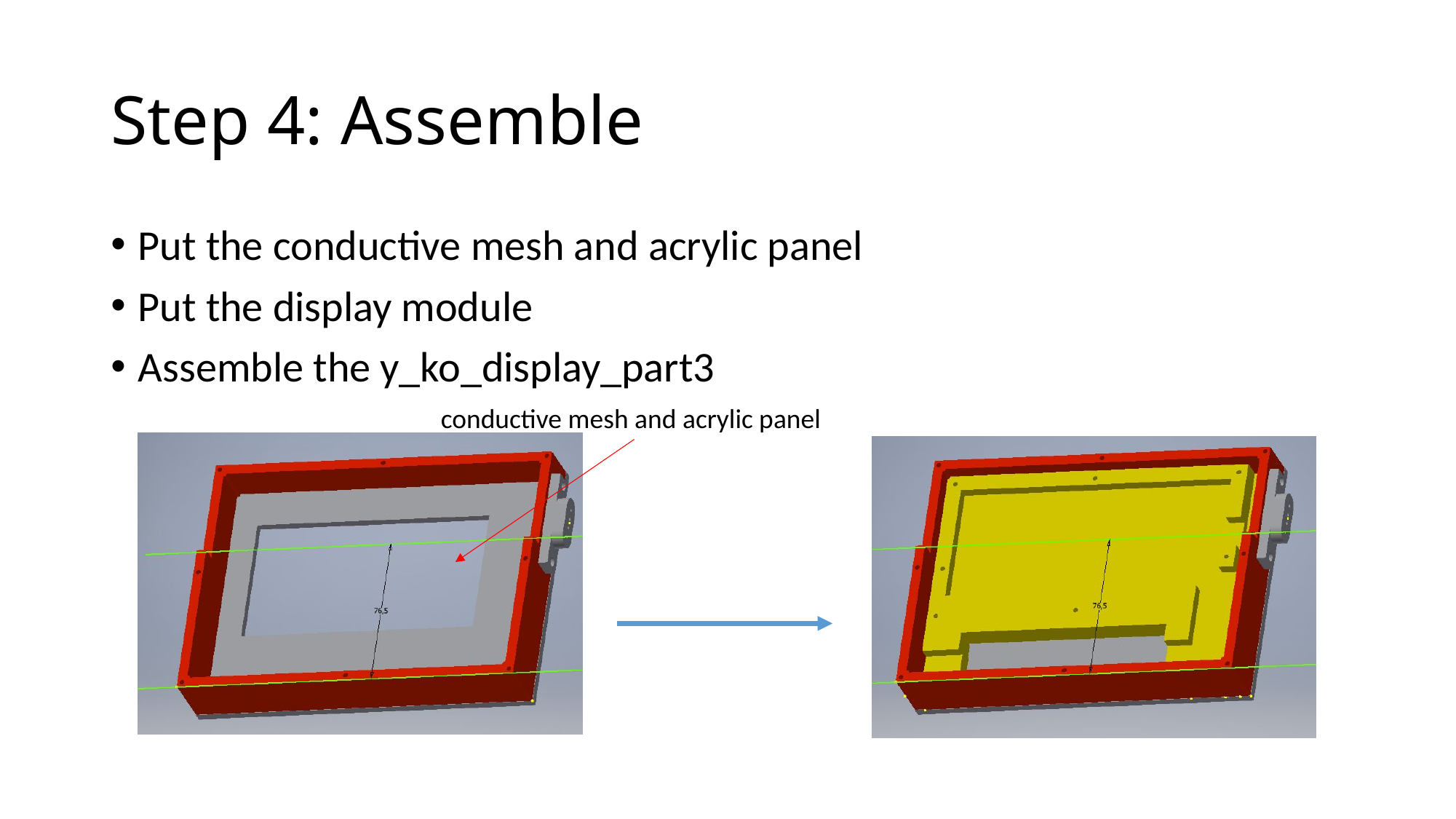

# Step 4: Assemble
Put the conductive mesh and acrylic panel
Put the display module
Assemble the y_ko_display_part3
conductive mesh and acrylic panel

## Slide 7
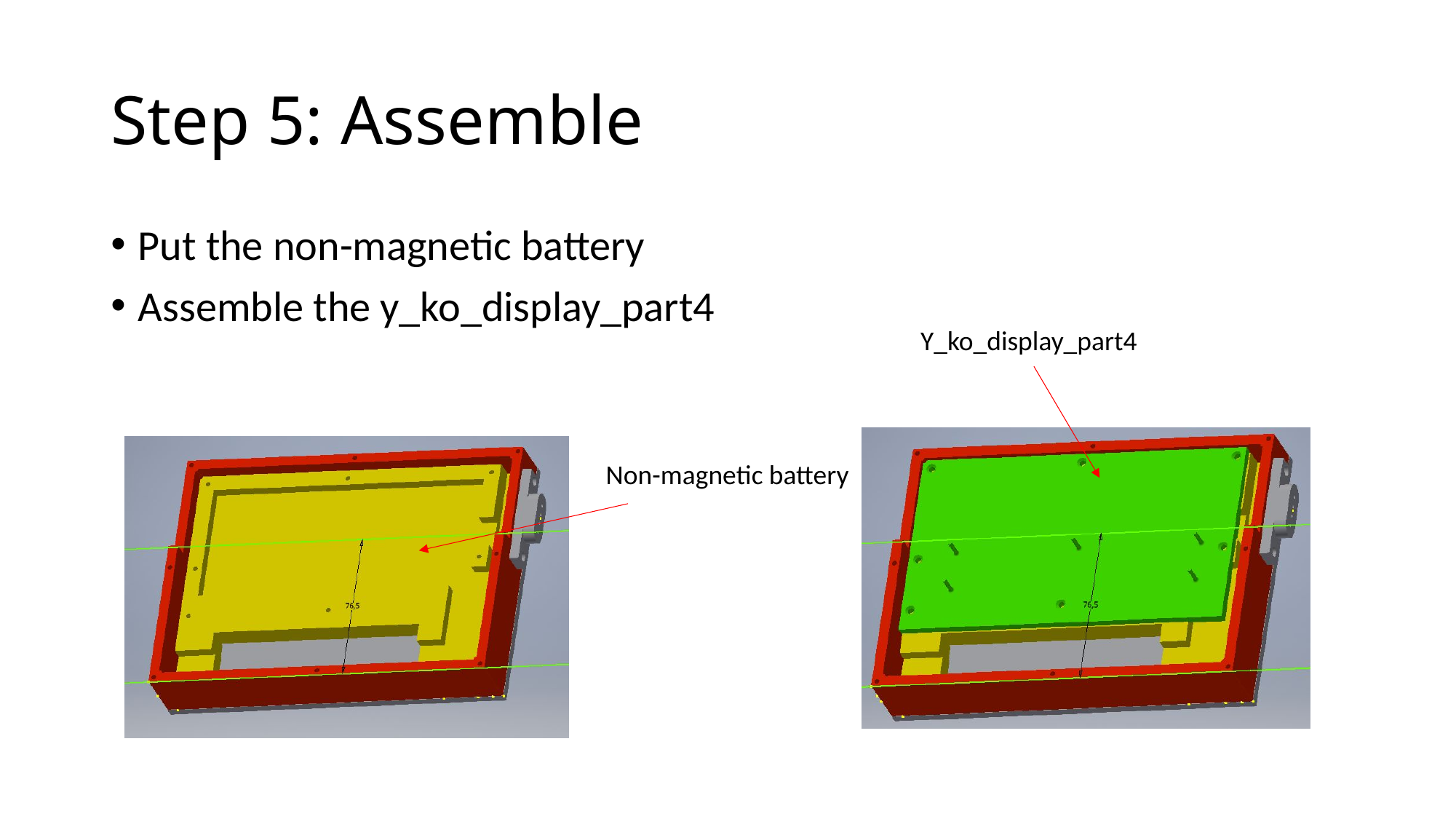

# Step 5: Assemble
Put the non-magnetic battery
Assemble the y_ko_display_part4
Y_ko_display_part4
Non-magnetic battery

## Slide 8
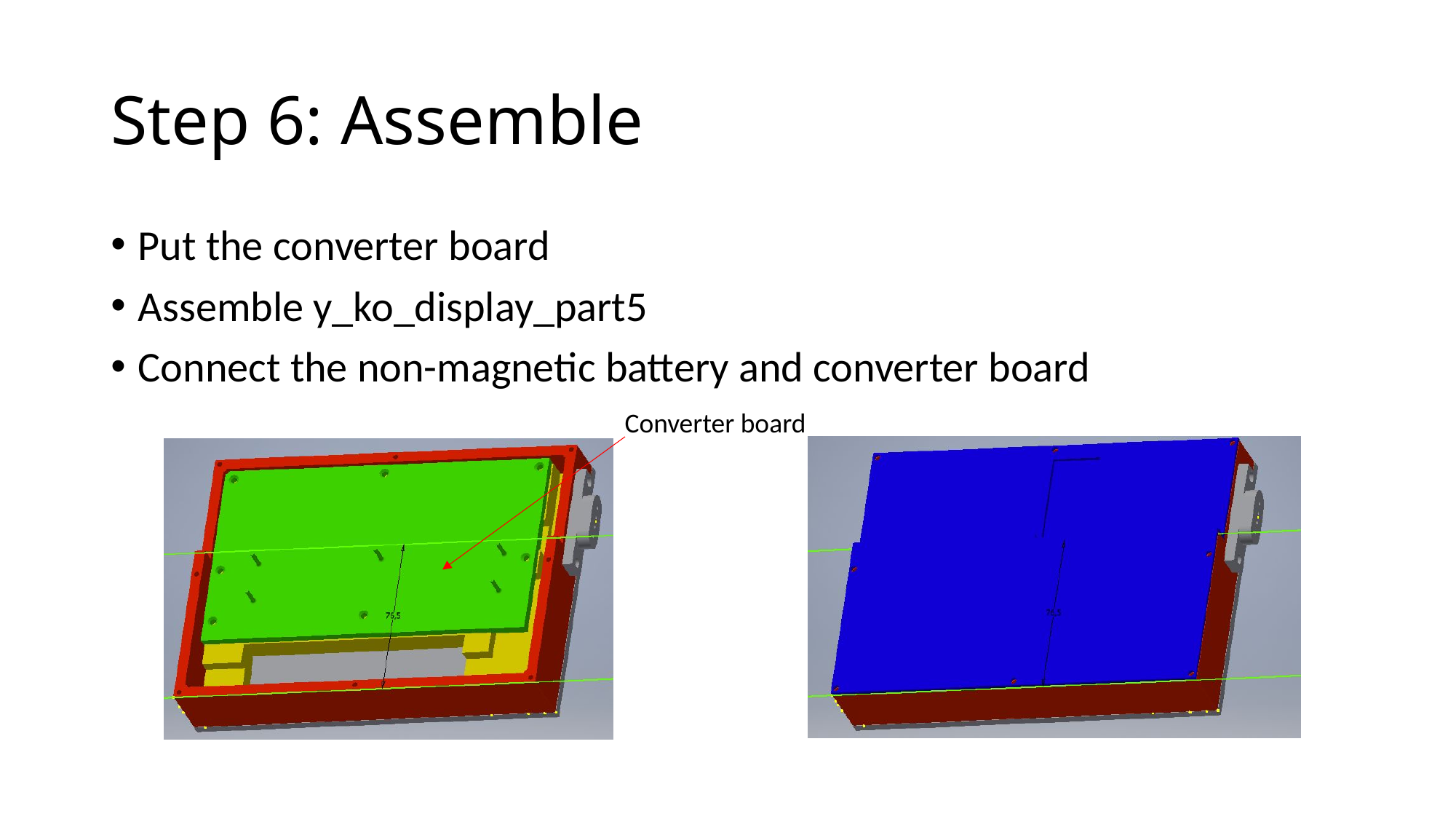

# Step 6: Assemble
Put the converter board
Assemble y_ko_display_part5
Connect the non-magnetic battery and converter board
Converter board

## Slide 9
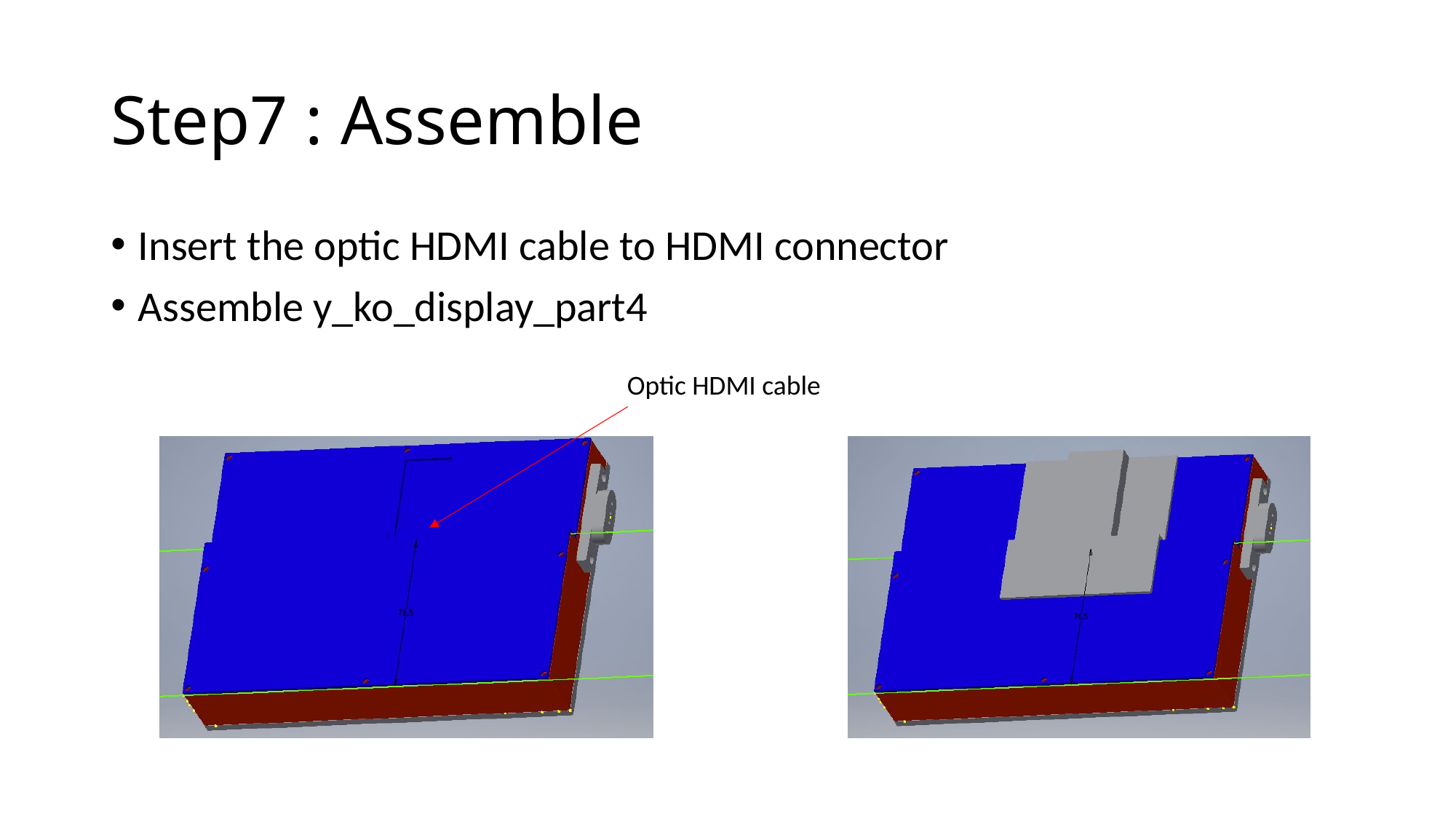

# Step7 : Assemble
Insert the optic HDMI cable to HDMI connector
Assemble y_ko_display_part4
Optic HDMI cable
